# Structural and Policy Determinants of Access to Medications for Opioid Use Disorder Among Pregnant People in U.S. Jails

**DOI:** 10.3390/ijerph23020149

**Published:** 2026-01-24

**Authors:** Maya Lakshman, Sitara Murali, Camille T. Kramer, Carolyn B. Sufrin, Rebecca L. Fix

**Affiliations:** 1 Department of Mental Health, Johns Hopkins Bloomberg School of Public Health, Baltimore, MD 21231, USA; smurali5@alumni.jh.edu (S.M.); csufrin@jhmi.edu (C.B.S.); rebecca.fix@jhu.edu (R.L.F.); 2 Department of Gynecology and Obstetrics, Johns Hopkins School of Medicine, Baltimore, MD 21224, USA; ckramer@jhu.edu

**Keywords:** medications for opioid use disorder, pregnancy, perinatal substance use, jails, correctional health, telemedicine, opioid treatment programs, maternal health systems

## Abstract

**Highlights:**

**Public health relevance—How does this work relate to a public health issue?**

- Pregnant people in U.S. jails experience elevated rates of opioid use disorder, yet access to evidence-based medications for opioid use disorder during incarceration remains inconsistent.
- This study examines how jail policies, treatment infrastructure, and political context shape access to perinatal OUD treatment in carceral settings.

**Public health significance—Why is this work of significance to public health?**

- Using national jail data, this analysis identifies modifiable structural and policy factors, such as telemedicine capacity and community provider availability, associated with MOUD provision during pregnancy.
- Findings extend prior descriptive work by quantifying system-level drivers of treatment access for a high-risk, underserved perinatal population.

**Public health implications—What are the key implications or messages for practitioners, policy makers, and/or researchers?**

- Expanding telemedicine and strengthening community MOUD treatment networks may substantially improve continuity of perinatal care for incarcerated pregnant people.
- Standardizing jail policies around perinatal MOUD provision is critical to advancing health equity and aligning correctional practice with public health and clinical guidelines.

**Abstract:**

Pregnant people in U.S. jails experience high rates of opioid use disorder (OUD), yet access to medications for opioid use disorder (MOUD) remains inconsistent. This mixed-methods study examines how jail policies, treatment infrastructure, and political context shape MOUD provision for pregnant incarcerated individuals. We conducted a secondary analysis of a national survey of 2885 U.S. jails (analytic sample = 836). Logistic regression models assessed associations between MOUD provision and telemedicine capacity, community MOUD availability, state Medicaid expansion, and 2020 presidential voting outcomes. Qualitative responses characterized barriers to care. Findings confirm that MOUD access for pregnant incarcerated individuals remains limited and structurally patterned. Fewer than half of jails continued methadone or buprenorphine for pregnant individuals already in treatment, and initiation was uncommon. MOUD provision was more likely in Democrat-won states, jails with telemedicine capacity, and jails located in communities with MOUD providers, while limited community availability reduced odds of provision. Qualitative themes highlighted restrictive jail policies, provider discretion, diversion concerns, and misconceptions regarding fetal harm. These findings underscore persistent structural barriers to evidence-based perinatal OUD treatment in carceral settings and highlight the importance of telemedicine expansion, community treatment capacity, and standardized correctional policies to advance perinatal health equity.

## 1. Introduction

Substance use during pregnancy remains a critical public health concern, with opioid exposure associated with neonatal opioid withdrawal syndrome, maternal morbidity, and pregnancy-associated overdose [1,2,3]. Medications for opioid use disorder (MOUD)—methadone and buprenorphine—are the evidence-based standard of care during pregnancy and reduce relapse, overdose, and adverse neonatal outcomes [2]. Yet access to MOUD in the United States remains uneven, particularly for people navigating the criminal legal system, fragmented care networks, and stigma [4].

The U.S. incarcerates nearly two million people across prisons, jails, juvenile detention, and immigration facilities [5], with a large proportion held in local jails [6]. Jails are transient institutions at the nexus of community and carceral health, characterized by high turnover, short stays, and under-resourced medical infrastructure [7]. Among people in jails, 63% meet criteria for drug dependence (72% of female adults, 62% of male adults) and roughly 400,000 people with OUD are arrested each year [7,8]. An estimated 3% of those entering jail are pregnant, and approximately 14% have OUD [9,10]. Because OUD is a chronic, treatable condition, MOUD is protected under the Americans with Disabilities Act, and constitutional standards requiring care for “serious medical needs” [11,12], denial or interruption of treatment during incarceration creates preventable medical and legal risk. This convergence of opioid use disorder, pregnancy-related health risk, incarceration, and stigma reflects a syndemic, in which co-occurring conditions interact synergistically under conditions of social and structural inequality to worsen health outcomes [13,14].

Despite clinical consensus, MOUD access in jails remains highly variable [10,15]. Recent federal policy changes have expanded pathways for treatment delivery—including removal of the buprenorphine X-waiver [16], allowance for methadone provision when jails register as hospitals or clinics [17], and expanded telemedicine and take-home authorization under 42 CFR Part 8 [18]. However, implementation has been inconsistent. While the National Commission on Correctional Health Care can certify opioid treatment programs (OTPs) in jails, accreditation is voluntary and does not extend to enforcement of MOUD provision [19,20]. As a result, most jails rely on strained partnerships with community OTPs, which are unevenly distributed and particularly scarce in rural regions [20,21].

These patterns are consistent with the concept of structural violence, which describes how social, political, and economic systems systematically produce harm by constraining access to resources, care, and safety for marginalized populations [22,23]. Because incarceration is geographically and demographically patterned, Black and Indigenous women—who are disproportionately jailed and have the highest maternal mortality rates [4,9]—are more likely to be detained in jurisdictions with fewer MOUD providers, limited Medicaid coverage, and weaker treatment infrastructure [24]. From an intersectional perspective, pregnancy, criminal legal involvement, race, and disability status related to OUD intersect to intensify exposure to structural barriers, producing distinct and compounded risks [25]. Thus, variation in state political orientation, telemedicine availability, and community MOUD capacity likely functions not only as administrative difference but as a racialized determinant of maternal health access.

A survey of nearly 3000 jails comprising the 2019 *National Jails Compendium* provided critical data on selected pregnancy care provision, including MOUD [26]. Using this resource, prior national work estimated that 60% of jails continue pre-incarceration pregnancy MOUD and only one-third of jails provide access to initiating MOUD in pregnancy [10,15]. However, the structural and policy forces that drive this variation remain insufficiently examined.

Further, this secondary analysis of a national jail survey tested predictors of MOUD provision. Specifically, we evaluated how policy and structural determinants, such as state political context, telemedicine adoption, Medicaid coverage, and community MOUD infrastructure, shape access to methadone and buprenorphine for pregnant individuals in jails. From this, we derived two specific research questions and corresponding hypotheses:

**RQ1.**  *Which structural, policy, and treatment-infrastructure characteristics are associated with MOUD provision for pregnant incarcerated individuals?*

**H1a.**  *Jails located in Medicaid expansion states and Democratic-won states (2020 election) would be significantly more likely to provide MOUD.*

**H1b.**  *Jails with telemedicine capacity and stronger community MOUD infrastructure (e.g., OTP availability, buprenorphine provider presence) would have significantly greater odds of MOUD provision.*

**H1c.**  *Jails reporting lack of community MOUD providers would be significantly less likely to provide treatment, reflecting dependency on external systems.*

**RQ2.**  *What barriers and challenges do jails report in delivering MOUD to pregnant individuals?*

**H2.**  *Qualitative analyses would reveal barriers primarily related to stigma, provider discretion, and misconceptions about fetal safety, as well as structural challenges such as staffing limitations and regulatory complexity.*

By integrating multilevel policy indicators with qualitative accounts from carceral health systems, this study identifies how structural determinants (as opposed to individual clinical judgment alone) shape maternal treatment access in jails, advancing efforts to align correctional health practice with federal disability protections and maternal health equity.

## 2. Methods

### 2.1. Participants

This is a secondary analysis of a cross-sectional survey administered to all 2885 US jails verified by the National Jails Compendium [27] between August and November of 2019 [15]. We requested a staff member at each jail who could report information on medications for opioid use disorder services and pregnancy care in their jail complete the survey. This was typically someone in leadership or the jail’s medical provider, although only 20% of the respondents were from the medical profession [15]. The *institution name redacted* Institutional Review Board (IRB) approved the study.

### 2.2. Survey Instrument

The 49-item instrument focused on methadone and/or buprenorphine availability and access for the treatment of pregnant people with OUD. For more details about the full survey design and implementation details, please see [10,15].

### 2.3. Measures

For this secondary data analysis, our primary outcome was composite MOUD provision, defined as whether a jail provided either continuation or initiation of methadone or buprenorphine to pregnant individuals (coded 0 = no MOUD of any kind; 1 = continuation or initiation of MOUD). Aligned with prior carceral MOUD literature, our secondary outcomes of interest were continuation and initiation of methadone/buprenorphine in the jail [10,15]. These items had been coded as (0 = no continuation; 1 = continuation) and (0 = no initiation; 1 = initiation) based on whether the jail allowed pregnant incarcerated individuals to continue and initiate MOUD in jail. Jails reporting provision of naltrexone were excluded from the analytic sample because naltrexone is not recommended for use during pregnancy [12].

Explanatory variables included the jails’ geographical classification; use of telemedicine (for any clinical service); use of behavioral treatment; and presence of MOUD providers in the community. Classifications were derived directly from survey items. Respondents indicated whether their jail served an urban, rural, or mixed jurisdiction; whether any telemedicine services were used; and whether behavioral treatment for opioid use disorder was offered. These responses were coded as urban (1), rural (2), or both (3); no telemedicine (0) or telemedicine use (1); and no behavioral treatment (0) or behavioral treatment provision (1). We coded jails that indicated if there is a place in their county for non-incarcerated individuals to receive methadone (1) or not (0), and to receive buprenorphine (1) or not (0). And lastly, we coded jails that indicated if a primary challenge was not having an MOUD provider in the community (1) or if this was not a challenge (0).

We incorporated additional explanatory variables using publicly available data, including 2020 state political context, Medicaid expansion status, and community MOUD availability. We defined 2020 state political context as the presidential voting outcome in the 2020 election, coding states won by the Republican candidate (“Republican-won states”) as (0) and states won by the Democratic candidate (“Democratic-won states”) as (1). Medicaid expansion was coded as (0) for states that had not adopted expansion and (1) for states that had adopted expansion at the time of data collection. Community availability of MOUD was assessed using the number of state opioid treatment programs (OTPs) listed in the SAMHSA directory [19]. OTPs were defined as clinics certified to dispense methadone, buprenorphine, and naltrexone for OUD treatment in the community [19].

### 2.4. Statistical Analysis

As previously reported in the parent study, 1139 unique survey responses were received from 2885 distributed surveys (39.5% response rate) [15]. The original analysis excluded responses that were inapplicable to jail settings (e.g., facilities housing only male adults), incomplete, or returned due to outdated addressee information [15]. For the current secondary analysis, we examined the remaining 836 eligible jail responses. We used descriptive statistics and chi-squared tests to summarize jail characteristics and examine bivariate associations with MOUD provision during pregnancy. The descriptive results presented in the Results Section are quantitative summaries of jail-level characteristics (e.g., MOUD provision, telemedicine capacity, community treatment availability) and do not involve individual participants. We then estimated univariate binary logistic regression models to assess the association between each explanatory variable and MOUD provision. Variables that were statistically significant in univariate analyses were entered simultaneously into a multivariable binary logistic regression model. The final multivariable model adjusted for jail-level characteristics (geographic classification and telemedicine use), state policy context (Medicaid expansion status and 2020 presidential voting outcome), and community treatment infrastructure (presence of community methadone and buprenorphine providers and reported lack of local MOUD providers). Because these factors are interrelated in practice, we anticipated overlap among explanatory variables; all were therefore included simultaneously in the multivariable models so that estimated associations reflect adjusted relationships independent of correlated policy and infrastructure factors. Odds ratios therefore represent the adjusted association between each predictor and the likelihood that a jail provided continuation or initiation of methadone or buprenorphine to pregnant incarcerated individuals, holding all other variables in the model constant. All analyses were conducted using SPSS version 28, and statistical significance was assessed at *p* < 0.05 [28].

### 2.5. Qualitative Analysis

The survey included two open-ended items: one asking respondents from jails why methadone or buprenorphine were not provided, and another asking them to describe challenges encountered when caring for pregnant individuals with OUD. We conducted a qualitative content analysis of these two text response survey items. Because qualitative data were collected via open-ended survey items, and our unit of analysis was each jail facility, quotations are attributed to jail facilities rather than to individual respondents. After reviewing these text responses, two members of our team developed a codebook, and then applied codes to the qualitative excerpts and grouped these codes by theme. These items yielded 17 codes for Item 1 and 16 codes for Item 2. SM and RF worked to ensure interrater reliability for coding. We utilized a final codebook with definitions and key quotes to summarize findings.

## 3. Results

As shown in Table 1, more than half of the jails in our sample were in Republican-won states (57.1%) and in states that had expanded Medicaid (56.8%). Many jails provided behavioral health treatment (72.7%), yet fewer than half used telemedicine (34.4%). Less than half of jails reported a presence of MOUD (42.7% for both methadone and buprenorphine) in the community for non-incarcerated individuals. The number of opioid treatment programs (OTPs) in states represented by respondents ranged from 0 to 168 (average: 54.7). Additionally, most respondents (577 jails, 69.3%) reported experiencing barriers to providing MOUD to pregnant incarcerated individuals, most frequently citing financial cost (34.3%), concerns about medication diversion (37.1%), and prescribing restrictions or regulatory requirements from the Drug Enforcement Administration (25.5%).

### 3.1. Factors Associated with MOUD Provision

We utilized multivariate logistic regression to determine which characteristics were associated with MOUD provision for pregnant incarcerated individuals in the sample jails (Table 2). In these models, odds ratios greater than 1 indicate higher odds that a jail provides methadone or buprenorphine to pregnant individuals compared with jails without that characteristic, after adjustment for other jail-, community-, and state-level factors. For example, jails located in Democratic-won states had nearly three times the odds of providing MOUD during pregnancy compared with jails in Republican-won states (OR = 2.830, 95% CI [1.955, 4.097], *p* < 0.001), controlling for other covariates. Similarly, jails using telemedicine had approximately 56% higher odds of providing MOUD than those without telemedicine capacity (OR = 1.562, 95% CI [1.115, 2.190], *p* = 0.010), net of other covariates.

Community treatment infrastructure was also strongly associated with MOUD provision. Jails located in communities with methadone availability (OR = 1.775, 95% CI [1.220, 2.582], *p* =0.003) or buprenorphine availability (OR = 2.387, 95% CI [1.651, 3.450], *p* < 0.001) had substantially higher odds of providing MOUD during pregnancy. In contrast, jails reporting concern about a lack of community MOUD providers had significantly lower odds of MOUD provision (OR = 0.601, 95% CI [0.389, 0.927], *p* = 0.021), underscoring the dependence of jail-based treatment on surrounding community infrastructure. In contrast, Medicaid expansion status was not significantly associated with MOUD provision in the adjusted models, and other tested jail-level characteristics did not reach statistical significance after adjustment (Table 2).

### 3.2. Barriers and Challenges to MOUD in Jails

As shown in Tables S1 and S2, jails reported multiple, often overlapping barriers that limited their ability to provide medications for opioid use disorder (MOUD) to pregnant incarcerated individuals. In Table S1 the most frequently reported barriers included physician discretion (27.5%), restrictive jail policies prohibiting MOUD (22.9%), and having very few pregnant individuals with OUD in custody (15.8%). In many facilities, MOUD availability depended largely on the medical director’s personal judgment rather than standardized clinical criteria. For example, another facility stated it would continue “the methadone regimen … until the inmate is able to be released,” while another stated that “the medical provider does not utilize other drugs to treat drug habit … unless it is critical [or] life-threatening.” This reliance on individual judgment often intersected with formal jail policy, as stated bluntly, “No narcotics are given at our jail.” Another facility explained, “We don’t get many if any pregnant women going through withdrawals in our jail.” These accounts illustrate wide variation in medical decision-making and inconsistency in applying evidence-based practice. Additional barriers jails reported facing included limited or no treatment availability (4.8%), lack of a licensed prescriber (5.5%), and insufficient staffing capacity (3.2%). Several of these themes also appeared in Table S2 featuring challenges jails reported more generally with pregnant individuals with opioid use disorder, where commonly reported challenges included having very few pregnant individuals requiring MOUD (18.1%) and, conversely, reporting no challenges at all (17.8%). One of the jails that reported no challenges expressed, “No current challenges exist once we contracted with local treatment center and our provider became certified to administer buprenorphine.”

Some other frequently reported challenges included perceptions of incarcerated individuals’ behavior (14.2%), other miscellaneous challenges related to treatment in jail (10.7%). For example, as explained by one facility, “Dispensing licenses for physicians are not easy to obtain and make it harder to obtain the ability to dispense MAT protocols in the jail.” Although less common, attitudinal and knowledge-based concerns were also identified. Some facilities reflected staff skepticism and mistrust of incarcerated individuals (2.2%), with reports of perceived medication misuse, resistance to treatment, or doubt regarding symptom legitimacy, including statements such as incarcerated individuals “faking symptoms to obtain a release from jail” or being labeled by staff as “habitual offenders” or “addicts.” Others expressed misconceptions about treatment safety during pregnancy, including concerns about fetal harm (6.4%) and complications in high-risk pregnancies (5.0%). Another facility stated that incarcerated individuals “hold baby ‘hostage’ regarding accepting MAT and/or prenatal medical care… [they] don’t want to harm the fetus,” while another asserted that detoxification during pregnancy was preferable despite clinical guidance to the contrary.

Resource limitations compound attitudinal barriers. Jails frequently cited staffing and licensure constraints, with one jail reporting simply, “No medical dept in jail,” and another noting that their physician “does not hold a DEA-Z number.”

Other less frequently reported challenges included lack of relevant treatment services within the jail (7.5%), difficulty ensuring continuation of MOUD following release (2.1%), and early release of pregnant individuals (1.9%). As one facility noted, “Rural community, lack of resources”. Another shared, “Lack of access to resources after release from jail and lack of source support after release from jail” made continuity of treatment difficult. Collectively, these findings suggest that barriers fall across intersecting domains of policy restrictions, clinical discretion, workforce capacity, population size considerations, stigma and misinformation, and gaps in continuity-of-care planning.

## 4. Discussion

Perinatal substance use remains a critical public health challenge that bridges clinical care, structural inequities, and social policy. Untreated opioid use disorder (OUD) during pregnancy is associated with elevated risks of relapse, overdose, and adverse birth outcomes [1,2,3], yet treatment access continues to lag need, particularly in structurally marginalized populations [27,29,30]. Recent work synthesizing jail- and prison-based medications for opioid use disorder (MOUD) programs highlights persistent gaps in implementation despite strong evidence of benefit [31]. Jails represent one of the most overlooked settings in this continuum. Each year, tens of thousands of pregnant people cycle through local jails, many with untreated OUD, yet few facilities provide consistent, evidence-based care [15,32]. Understanding how jail policies, provider availability, and broader political environments shape access to MOUD is essential for advancing maternal and infant health equity.

Consistent with our hypotheses, jails located in Democratic-won states and those within states that expanded Medicaid were significantly more likely to offer MOUD. This pattern aligns with prior research showing that state policy environments and political orientation influence the accessibility of addiction treatment [27,33]. Importantly, we anticipated substantial overlap among several predictors examined in this study. States with Democratic presidential voting outcomes are more likely to have expanded Medicaid, greater community MOUD treatment capacity, and stronger public health infrastructure overall. By including these variables simultaneously in the multivariable models, we were able to estimate the independent association of each factor with MOUD provision in jails, net of correlated policy and treatment environment characteristics. The persistence of significant associations after adjustment suggests that political context, telemedicine capacity, and community treatment availability each contribute distinct—though interconnected—pathways shaping access to perinatal MOUD in carceral settings. Notably, our sample included an almost even distribution of jails located in Democratic-won versus Republican-won states, which strengthens confidence that these associations are not simply artifacts of sampling imbalance. Rather, the nearly 50/50 split suggests that political context meaningfully shapes treatment access even when jails across the political spectrum are well represented. Importantly, even in states with laws mandating MOUD access in jails, implementation remains inconsistent and incomplete [34]. Because the ADA classifies OUD as a disability, jails that prohibit continuation of methadone or buprenorphine for individuals already receiving treatment may be in violation of federal law [11,34]. Greater oversight by the Department of Justice is needed to ensure compliance and to close the gap between policy intent and carceral practice.

Telemedicine capacity emerged as a key factor associated with MOUD provision. Although only one-third of jails in our sample reported any telemedicine use, those that did were significantly more likely to offer methadone or buprenorphine. This aligns with research demonstrating that telemedicine increases access to OUD care and maintains comparable clinical outcomes to in-person treatment [35,36]. Temporary SAMHSA flexibilities introduced during the COVID-19 pandemic (e.g., allowing take-home methadone doses and buprenorphine initiation via telemedicine) proved effective and did not increase diversion or overdose risk [18,37]. These changes demonstrated that lower-burden, patient-centered approaches to MOUD delivery can be both safe and clinically effective. Recent federal guidance further expands access: under updated SAMHSA regulations, pregnancy now qualifies as a pre-existing medical condition that allows correctional facilities to dispense methadone if they register as a hospital or clinic rather than a full opioid treatment program (OTP). When combined with the elimination of the buprenorphine X-waiver under the 2023 MATE Act [16], these policy shifts provide new mechanisms for jails—especially those located in rural areas—to expand onsite MOUD. However, these regulatory changes are still not widely known or consistently implemented, suggesting an urgent need for technical assistance and dissemination targeted to correctional systems.

Community treatment capacity emerged as another strong predictor of MOUD access. Jails located in states with more opioid treatment programs (OTPs) and buprenorphine prescribers had substantially higher odds of providing MOUD onsite. This likely reflects structural dependence on community systems: most jails do not operate in-house OTPs and therefore rely on external clinics for dosing, medication transport, and prescriber authorization [38]. In rural jurisdictions, where OTPs are scarce and travel distances are significant, jails may be unable to arrange daily methadone transport or coordinate buprenorphine initiation [39]. Our findings demonstrate that MOUD provision in jails is not determined solely by correctional policy but by the treatment ecosystem surrounding the facility. In other words, jails can only provide what their local health infrastructure makes possible, reinforcing geographic inequities in pregnancy care access.

Our qualitative findings help explain why these structural opportunities have not yet been translated into universal access. Many jails reported that MOUD decisions depended solely on physician discretion or restrictive jail policies, leading to inconsistent and sometimes medically unsound practices. Staff narratives reflected persistent stigma toward incarcerated pregnant individuals, including misconceptions that MOUD constitutes “drug seeking” or causes fetal harm; these beliefs are contradicted by clinical evidence [40,41]. Such attitudes mirror the experiences described by incarcerated pregnant people themselves. Many pregnant individuals with OUD face dehumanization, withdrawal suffering, and punitive treatment when in jail [29]. Complementary interviews with community opioid treatment providers highlight additional barriers, such as fragmented coordination with jails, distrust from jail staff, and difficulties maintaining treatment across custody transitions [42]. Together, this literature underscores that stigma and carceral culture—rather than clinical uncertainty—often drive decisions to withhold evidence-based care.

Resource limitations compound these attitudinal barriers. Respondents cited the lack of licensed prescribers, staffing shortages, and limited proximity to opioid treatment programs (OTPs) as major obstacles to care, again particularly impacting facilities in rural jurisdictions [43,44]. Expanding OTP capacity, leveraging telemedicine networks, and extending Medicaid coverage for incarcerated populations, as recently approved in California’s Section 1115 waiver demonstration [45], are promising approaches to improving continuity of care during and after incarceration. These efforts could reduce postpartum relapse, overdose, and recidivism, which remain high among justice-involved populations [46].

Incarcerated individuals are the only population in the U.S. with a constitutional right to healthcare, yet the quality and consistency of that care often fall far short of clinical and ethical standards [34]. National guidelines from ACOG and SAMHSA support methadone and buprenorphine as first-line treatments for OUD during pregnancy because they are safe, effective, and protective against overdose and neonatal morbidity [30]. Despite these recommendations, as reported in a previous publication with these data, fewer than half of U.S. jails provide continuation of either medication for pregnant individuals; even fewer initiate treatment while in jail [15]. Every jail in our sample reported having at least one pregnant person, underscoring that the population in need is widespread.

The absence of standardized, evidence-based perinatal treatment in jails perpetuates both structural and reproductive inequities. Black women are incarcerated at nearly three times the rate of white women, and the harms of untreated OUD—poor maternal outcomes, neonatal complications, and postpartum relapse—disproportionately affect Black and Indigenous families [47]. Together, these findings demonstrate how structural violence, syndemic conditions, and intersecting systems of disadvantage shape access to perinatal OUD treatment in jails, moving beyond individual-level explanations toward policy-relevant structural determinants. Addressing these inequities requires systemic reforms that integrate correctional, clinical, and public health infrastructures.

## 5. Limitations and Future Directions

This study has several limitations. First, the survey relied on voluntary participation, introducing potential self-selection bias, and all responses were self-reported, which may limit accuracy. Second, the data were collected in 2019, before major federal regulatory changes related to methadone, buprenorphine, and telemedicine, including COVID-era flexibilities and subsequent policy updates. As a result, MOUD availability and practices in jails may have evolved since the time of data collection. Updated national surveys of U.S. jails are needed to assess whether and how these post-2019 regulatory changes have translated into expanded access to perinatal MOUD in carceral settings. Third, because the survey was cross-sectional, it could not capture temporal changes in facility policies or responses to this evolving regulatory landscape. Longitudinal or repeated cross-sectional studies would allow future research to examine how shifts in federal and state policy environments shape MOUD availability over time, rather than relying on single snapshots. Fourth, some classifications—such as urban versus rural jurisdiction—were based solely on respondents’ self-reported descriptions rather than standardized geographic definitions, introducing potential subjectivity and misclassification.

Qualitative free-text responses were optional and varied widely in depth and detail, limiting generalizability. Future surveys should incorporate structured response options to better capture the range and frequency of challenges across facilities. Moreover, the survey did not collect demographic information on incarcerated individuals, preventing examination of inequities by race, ethnicity, gender identity, or other social identifiers. Given the disproportionate incarceration of Black, Indigenous, and other people of color in the U.S., future research should explicitly assess how racialized and gendered dynamics shape access to perinatal addiction treatment. Integrating the perspectives of incarcerated individuals through qualitative interviews would further strengthen understanding of both structural and interpersonal barriers to MOUD.

## 6. Conclusions

This secondary analysis of 836 U.S. jails extends prior to national estimates by identifying which and how structural, political, and treatment-infrastructure factors most strongly predict MOUD provision during pregnancy. Our findings underscore key opportunities to advance perinatal health equity within correctional settings. Expanding telemedicine infrastructure, increasing funding for community and jail-based opioid treatment programs, and improving staff education on the safety and efficacy of MOUD during pregnancy could substantially reduce preventable morbidity and mortality. Oversight and enforcement from federal agencies such as the Department of Justice, the National Commission on Correctional Health Care, and the Substance Abuse and Mental Health Services Administration (SAMHSA) are essential to ensure compliance with national guidelines and the Americans with Disabilities Act. Ensuring that pregnant individuals receive evidence-based treatment for opioid use disorder while incarcerated is not only a public health priority but also a fundamental matter of reproductive and human rights. Addressing these systemic gaps is critical to improving maternal outcomes, reducing intergenerational harm, and advancing equity in carceral and community health systems alike.

## Figures and Tables

**Table 1:** Characteristics of Respondents in Jails.

Characteristic	*n*/Total *n* (%)
Total Respondents ^a^	836 (100.0)
Geographical Classification Urban Rural Both urban and rural Other	190/823 (23.1) 559/823 (67.9) 48/823 (5.8) 26/823 (3.2)
State Political Context Democratic-won Republican-won	358/834 (42.9) 476/834 (57.1)
State-Level Medicaid Expansion Status Expanded Not Expanded	474/834 (56.8) 360/834 (43.2)
Presence of Pregnant Incarcerated Individuals	831/831 (100)
Usage of Telemedicine in Jail	287/834 (34.4)
Availability of Behavioral Health Treatment	607/835 (72.7)
Presence of MOUD in the Community for Non-Incarcerated Individuals Methadone Buprenorphine	357/836 (42.7) 357/836 (42.7)
Provision of Methadone-Assisted Treatment	486/836 (58.1)
Continuation of MOUD Continue Methadone Continue Buprenorphine	375/836 (44.9) 387/836 (46.3)
Initiation of MOUD Initiate Methadone Initiate Buprenorphine	149/836 (17.8) 218/836 (26.1)
Top 3 Methods of Methadone Provision Pick Up from Community OTP Patients Transported to Community OTP Other	205/836 (24.5) 199/836 (23.8) 163/836 (19.4)
Top 3 Methods of Buprenorphine Provision Provided On-site by Jail Clinical Providers Patients Transported to Community Clinic Other	262/836 (31.3) 96/836 (11.5) 161/836 (19.3)
Jails Facing Challenges in Providing MOUD	577/833 (69.3)
Top 3 Concerns Facing Jails Regarding MOUD Provision Financial Cost Concerns Regarding Medication Diversion DEA and Prescribing Regulations	287/836 (34.3) 310/836 (37.1) 213/836 (25.5)
Number of OTPs in the State	Mean (SD) 54.7 (46.5)

^a^ Missing data were excluded from the results in Table 1 and percentage calculations. 13 respondents did not report their geographical classification, 2 did not report their state, 2 did not report their telemedicine usage, 1 did not report their behavioral treatment usage, 5 did not report their presence of pregnant incarcerated individuals, 3 did not respond to facing challenges in provision of MOUD.

**Table 2:** Geographical, Community, and Political Characteristics Associated with MOUD Provision for Pregnant Incarcerated Individuals.

Characteristics	OR (95% CI)	*p*-Value ^a^
Geographical Classification Rural vs. Urban Both Rural and Urban vs. Urban Other vs. Urban	0.833 (0.553, 1.255) 1.352 (0.629, 2.909) 1.940 (0.605, 6.221)	0.382
State Political Context Democratic-won vs. Republican-won	2.830 (1.955, 4.097)	<0.001 ***
Medicaid Expansion Status Expanded vs. Has Not Expanded	0.795 (0.554, 1.141)	0.213
Usage of Telemedicine in Jail Yes vs. No	1.562 (1.115, 2.190)	0.010 *
Behavioral Treatment in Jail Yes vs. No	1.225 (0.851, 1.763)	1.225
Methadone Treatment in the Community Yes vs. No	1.775 (1.220, 2.582)	0.003 **
Buprenorphine Treatment in the Community Yes vs. No	2.387 (1.651, 3.450)	<0.001 ***
No. of OTPs in the State	1.001 (0.998, 1.005)	0.523
Concerned about lack of MOUD Provider in the Community Yes vs. No	0.601 (0.389, 0.927)	0.021 *

^a^ Significance levels (* *p* ≤ 0.05; ** *p* ≤ 0.01; *** *p* ≤ 0.001).

## Data Availability

The data analyzed in this study were obtained from a national survey of U.S. jails conducted as part of a parent study. While the dataset is not directly archived by the authors, it may be available from the parent study investigators upon reasonable request and subject to any applicable data use agreements. Aggregated data supporting the findings of this study are included within the article.

## Supplementary Materials

The following supporting information can be downloaded at: https://www.mdpi.com/article/10.3390/ijerph23020149/s1, Table S1: Themes Appearing in Qualitative Analysis of Reasoning Why MOUD with Methadone or Buprenorphine Is Not Provided, Table S2: Themes Appearing in Qualitative Analysis of Challenges Jails Face with Pregnant Individuals with OUD.
