# Supplementary material for: Structural and Policy Determinants of Access to Medications for Opioid Use Disorder Among Pregnant People in U.S. Jails
Source: Int J Environ Res Public Health. 2026 Jan 24;23(2):149. doi: 10.3390/ijerph23020149 (PMC12940283; doi:10.3390/ijerph23020149)
Supplement: Supplementary file 1 [file ijerph-23-00149-s001.zip › ijerph-4071145-supplementary.pdf]

Table S1. Themes Appearing in Qualitative Analysis of Reasoning Why MOUD with Methadone or Buprenorphine Is Not Provided.

Theme	n (%)	Definition	Example Quote
Total Respondents [a]	495 (100)		
Doctor's discretion	136 (27.5)	Treatment is determined case-by-case and ultimate decision lies with patient's physician	"The Medical Director takes this on a case by case basis. Most pregnant women who are pregnant already have a methadone regimen established and the medical director will continue until the inmate is able to be released."<br><br>"The Jail's Medical Director states that [treatment] isn't necessary."<br><br>"Medical provider does not utilize other drug to treat a drug habit unless it is critical [or] life threatening."
Jail policy	113 (22.9)	Jail policy does not allow use of narcotics in facility; jail policy involves use of different treatment for withdrawal symptoms rather than MOUD	"No narcotics are given at our jail"
Doesn't apply	78 (15.8)	Jails who report not having many pregnant individuals with OUD in their facility or jails who reported they are providing MOUD, but only either methadone or buprenorphine	"We don't get many, if any, pregnant women going through withdrawals in our jail"<br><br>"Methadone not provided but Buprenorphine is"
Lack of provider	27 (5.5)	Facility physician lacks appropriate Drug Enforcement Agency licensing to provide MOUD or there is no physician available for prescribing treatment	"Facility physician does not hold a DEA-Z number. No outside source to come in house for treatment"
Other	27 (5.3)	Miscellaneous reasons why jail is not providing treatment	"We are only a temp-holding facility"<br>"Pharmacy is not licensed for this level of narcotics"
No/limited treatment available	24 (4.8)	Treatment is not provided due to lack of providers	"Our doctor can not prescribe. No Methadone clinic in county"

Only continue, do not initiate	23 (4.6)	Jail will not initiate treatment but will continue if individual arrives with prescription from the community	"If the inmate was already taking these medications, we would continue them. We would not start them. Medical provider uses other alternatives."
Program in process	18 (3.6)	Jail is working on initiating an MOUD program, but it has not yet started	"We just recently received the go-ahead to create an MAT program here."
Lack of staff	16 (3.2)	Lack of medical staff in the jail, such as nurses, to administer treatment	"No medical dept in jail"
External provision only	11 (2.2)	Individuals receive treatment outside of jail only	"Our provider is not licensed to write script for Buprenorphine or Methadone. Pregnant female is transported to facility for doctor to write script."
Taper off	9 (1.8)	Jail will taper individual off the MOUD they are taking prior to incarceration	"If an inmate arrives taking methadone, we do a wean off to discontinue."
Concern about misuse	7 (1.4)	Jail does not provide MOUD due to concern that incarcerated individuals will divert or abuse medication	"Due to the increased likelihood of drug diversion amongst inmates"
Lack of funding	6 (1.2)	Jail lacks funding to provide treatment	"The facility will not pay for it."
Concern about fetus	4 (0.8)	Jail is concerned that MOUD will harm the fetus, so they do not provide treatment	"It has not been proven to benefit…We believe the safest place for withdrawal of fetus along with any other fetal health issues is in the womb with the Mother under observation in a secure environment. We have been detoxing pregnant females from opiates and other drugs for years (15+). Babies are born healthy and not addicted to ANYTHING."<br><br>"We have had providers say to us that there is no way to monitor the fetus during the detox process and that's why they recommend continuing the use of these prescribed drugs during the pregnancy. We do not agree with this…"
Potential medical release	3 (0.6)	Individuals will potentially be released to a hospital or facility to determine treatment	"Its possible we would release on medical and let them get the help needed."
Only for pregnant people	3 (0.6)	MOUD is only provided on a case-by-case basis for pregnant people in the jail	"She's transferred to get treatment in pregnancy and is then tapered/returned once the pregnancy ends."
Unknown	3 (0.6)	Respondent did not know why treatment is not provided	"Unknown"

[a] Some responses were coded as part of one or more themes, so the totals for each theme do not add up to the total response 'n'. The percents for each theme were calculated by dividing the totals for each theme by the number of responses.

Table S2. Themes Appearing in Qualitative Analysis of Challenges Jails Face with Pregnant Individuals with OUD.

Theme	N (%)	Definition	Key Quote
Total Respondents [a]	535 (100)		
Doesn't apply	97 (18.1)	No challenges due to none or few pregnant incarcerated individuals with OUD in the jail	"We very seldom have anyone pregnant on MAT"
None	95 (17.8)	No current challenges	"No current challenges exist once we contracted with local treatment center and our provider became certified to administer buprenorphine."
Incarcerated individuals' behavior [b]	76 (14.2)	Challenges related to incarcerated individual's behavior in response to taking MOUD, subcategories listed below	"Inmates diverting medications"
Other	57 (10.7)	Miscellaneous challenges related to MOUD treatment in jail	"Dispensing licenses for physicians are not easy to obtain and make it harder to obtain the ability to dispense MAT protocols in the jail." "Methamphetamines is our major issue"
Transport	41 (7.7)	Transporting individuals to a clinic or hospital to receive treatment is challenging	"Getting pregnant females into inpatient facilities"
Lack of services	40 (7.5)	Lack of services includes limited resources for MOUD, prenatal care, and healthcare in general for incarcerated individuals	"Rural community, lack of resources" "Lack of or no prenatal care prior to incarceration. not knowing they are pregnant until they come to jail."
Fetal harm	34 (6.4)	Jail is concerned that MOUD will harm the fetus, so they do not provide treatment; Incarcerated individual is concerned that MOUD will harm the fetus, so they do not accept treatment	"Inmates hold baby 'hostage' in regards to accepting MAT and/or prenatal medical care. Don't want to harm fetus" "The direct effect opioid use has on the fetus is the greatest challenge."

Medication misuse	32 (6.0)	Challenges related to fear of incarcerated individuals diverting or abusing MOUD	"These patients are still addicts and continue with the behaviors. They attempt to save, store and share the medications. They also continue to seek higher and higher dosages. We are providing a metered high but are unable to do anything to change the behavior associated with the addiction."
High-risk pregnancy	27 (5.0)	Incarcerated individual's pregnancy is considered high-risk, which presents a challenge to providing MOUD or prenatal care	"Even a healthy pregnancy is a high-risk pregnancy for individuals that are incarcerated, so adding in SUD or OUD only increases these risks. Our medical team also considers this a high-risk population, and they will make the necessary arrangements in order to meet the inmate-patient's needs."
Withdrawal/detox	22 (4.1)	Challenges related to pain and discomfort from withdrawals or detox process	"Withdrawal stages and having the amount of staff for continuous monitoring"
Resistance to treatment	17 (3.2)	Challenges related to incarcerated individuals who do not want treatment for reasons such as fear of fetal harm and lack of knowledge around treatment	"Resistance from the inmate for treatment or help. Incredible amount of manipulative behavior"
Lack of staff	16 (3.0)	Lack of medical staff in the jail, such as nurses, to administer treatment	"Short staff and we do not have a medical personnel 24/7"
Lack of provider	14 (2.6)	Lack of physicians who can prescribe or access MOUD for patients	"Qualified medical providers willing to order MAT"
Lack of trust	12 (2.2)	Challenges related to jail staff mistrusting incarcerated individuals regarding drug usage or health symptoms	"Inmates faking/playing up symptoms to obtain a release from jail."<br><br>"They seem to be habitual offenders with little to no care for there [sic] unborn child, themselves or others."
Limited treatment available	11 (2.1)	Jail struggles to obtain MOUD due to limited availability of medication in the community	"Limited quantities of methadone available from community clinics. Jail doctor is not MAT certified"

Continuation after release	11 (2.1)	Continuing MOUD after release is challenging due to lack of resources or compliance	"Lack of access to resources after release from jail and lack of source support after release from jail."
Early release	10 (1.9)	Incarcerated individuals are given higher opportunity to be released, which may present challenges with MOUD	"This is addressed on a case by case basis by the medical provider. In most circumstances pregnant inmates are released from custody shortly after arrest and are not held in jail."
Funding	10 (1.9)	Jail does not have the funding to provide treatment	"Cost, transportation and no step-down program are factors."
Other drug usage	9 (1.7)	Concerns regarding polysubstance use by incarcerated individual that may impede treatment	"Possible use of other drugs in jail interacting with MAT"
Internal attributes	8 (1.5)	Challenges related to jail staff characterizing incarcerated individuals as unwilling to comply with treatment due to addiction and mental health	"They seem to be habitual offenders with little to no care for there [sic] unborn child, themselves or others."
General behavior	6 (1.1)	Miscellaneous challenges related to incarcerated individual's behavior	"Behavior management while withdrawing"
Doctor's discretion	5 (0.9)	Treatment is determined case-by-case and ultimate decision lies with patient's physician	"Medical providers are split as to whether MAT is better for the fetus than no MAT, and different doctors choose different options. There is no consistent protocol, which leads to "you treated so-and-so with drugs..."

[a] Some responses were coded as part of one or more themes, so the totals for each theme do not add up to the total response 'n'. The percents for each theme were calculated by dividing the totals for each theme by the number of responses.

[b] Incarcerated individuals' behavior was coded initially, and subcategories followed: general behavior, medication misuse, lack of trust, resistance to treatment, internal attributes, other drug usage.
